# Estrogen-Like Effect of Mitotane Explained by Its Agonist Activity on Estrogen Receptor-α

**DOI:** 10.3390/biomedicines9060681

**Published:** 2021-06-16

**Authors:** Elisa Rossini, Edoardo Giacopuzzi, Fabrizio Gangemi, Mariangela Tamburello, Deborah Cosentini, Andrea Abate, Marta Laganà, Alfredo Berruti, Salvatore Grisanti, Sandra Sigala

**Affiliations:** 1 Section of Pharmacology, Department of Molecular and Translational Medicine, University of Brescia, 25123 Brescia, Italy; e.rossini013@unibs.it (E.R.); m.tamburello@studenti.unibs.it (M.T.); a.abate005@studenti.unibs.it (A.A.); 2 Wellcome Centre for Human Genetics, University of Oxford, Oxford OX3 7BN, UK; edg1983@well.ox.ac.uk; 3 National Institute for Health Research Oxford Biomedical Research Centre, Oxford OX4 2PG, UK; 4 Unit of Physics, Department of Molecular and Translational Medicine, University of Brescia, 25123 Brescia, Italy; fabrizio.gangemi@unibs.it; 5 Medical Oncology Unit, Department of Medical and Surgical Specialties, Radiological Sciences, and Public Health, University of Brescia at A.S.S.T. Spedali Civili di Brescia, 25123 Brescia, Italy; deborah.cosentini@gmail.com (D.C.); martagana@gmail.com (M.L.); alfredo.berruti@gmail.com (A.B.); grisanti.salvatore@gmail.com (S.G.)

**Keywords:** estrogen receptor α, mitotane, adrenocortical carcinoma, bioinformatics analysis

## Abstract

Mitotane is the cornerstone of medical treatment of adrenocortical carcinoma. Estrogenic-like side effects frequently occur in patients, and previous studies explored the chemical nature of the interaction between estrogen receptor-α (ER-α) and toxic compounds, including the DDD derivatives. We used molecular docking and molecular dynamics (MD) simulations to explore the possible interaction between mitotane and the ER-α receptor and the induced conformational changes. The ER-α expressing MCF-7 cells were exposed to mitotane with/without tamoxifen, and the cell viability/proliferation was evaluated by MTT assay and direct count. The transient ER-α silencing was performed using two ER-α siRNA (50 nM) and verified by Western blot. MDA-MB-231 cells were used as a negative control. Mitotane showed a similar docking configuration to 17β-estradiol and bisphenol A (BPA) and a significant binding affinity to ER-α. MD simulations showed that mitotane preserves the active conformation of ER-α more than both BPA and Bisphenol C, classifying it as an agonist. Exposure of MCF-7 cells to mitotane led to the concentration-dependent increase of cell viability and proliferation, which was reduced in the presence of tamoxifen and nullified by the transient ER-α knock-down. Integrating bioinformatics approaches with cell biology and pharmacological methods, we demonstrated that mitotane directly binds and activates ER-α.

## 1. Introduction

Adrenocortical carcinoma (ACC) is a rare malignancy with a reported incidence of 0.7–2 cases per million population per year [1,2]. It is more frequent in females and can occur at any age, with a peak in the fifth and sixth decades of life. As a result of cancer-specific altered transcriptional modules [3], about 50–60% of ACCs are hormone functioning at presentation. Hypercortisolism (Cushing’s syndrome) or mixed Cushing’s and virilizing syndromes are observed in 50–80% of hormone-secreting ACCs [1,2]. In patients with resected ACC, the presence of clinical signs of cortisol excess is an independent poor prognostic factor [4].

Mitotane (o,p’-DDD) is the cornerstone of medical treatment of ACC, both in adjuvant [5,6] and metastatic settings [7]. The drug has selective cytotoxic activity against cells of the zona reticularis and zona fasciculata of the adrenal cortex, which leads to a marked inhibition of adrenocortical steroid synthesis, resulting in the control of hormone excess-associated syndromes [8]. Mitotane therapy invariably leads to adrenal insufficiency because of the functional suppression of the remaining normal adrenal gland, requiring chronic exogenous cortisol replacement therapy [1,2]. Therefore, patients with ACC receiving long-term mitotane face both direct (mitotane inhibition of steroidogenesis) and indirect (exogenous cortisol replacement therapy) endocrine effects.

One common clinical observation in patients undergoing mitotane administration is the high incidence of estrogenic-like side effects. Indeed, approximately one-third of adult male patients develop gynecomastia eventually associated with other signs of secondary hypogonadism such as hair loss, libido loss, muscle mass loss, and psychological alterations [9,10]. In premenopausal fertile women, mitotane induces the formation of benign ovarian cysts, amenorrhea, or abnormal uterine bleeding such as menorrhagia and metrorrhagia [11,12], and it has been associated with increased frequency of peripheral precocious puberty in female children [13].

Early studies demonstrated that most ACC patients treated with mitotane have increased levels of sex hormone-binding globulin (SHBG), a glycoprotein involved in binding and transport of circulating plasma testosterone and 17β-estradiol (E2) to target tissues [9,14]. In an in vitro study, Nader et al. demonstrated that mitotane increases gene expression of SHBG in an estrogen-receptor-α (ER-α)-dependent manner [15]. Thus, it has been postulated that at least some of the estrogen-like effects of mitotane therapy could be explained in terms of ER-α-dependent increase of SHBG and subsequent perturbation of bioavailability of plasma testosterone and E2 for target tissues.

ER-α and ER-β have been described in the normal adrenal cortex during fetal development and in postnatal life [16,17]. ERs are expressed at different intensities in both normal and neoplastic adrenal cortices [17]; however, the pathophysiological relevance of their expression in adrenal cell proliferation is not completely elucidated. The ER-α subtype appears to be generally poorly expressed, while ER-β is expressed mainly in the glomerular and fasciculata areas [18,19]. Data concerning their expression in ACC are controversial, although results obtained with immunohistochemical analyses indicated that ER-α expression is weak/undetectable [17,20]. Contrasting results suggest indeed low ER-β and/or high ER-α expression in ACC, leading to an increase in the ER-α/ER-β ratio, compared to that observed in healthy tissue [19].

Previous studies suggested that mitotane could bind the human ER-α with a relative binding affinity of about 1000-fold weaker than the natural ligand, E2 [21,22,23]. However, these studies mainly explored the chemical nature of the interaction between ER-α and potential environmental toxic compounds.

At physiological conditions, ER-α exists as a dimer and can present itself in different conformations stabilized by the binding of either agonists or antagonists [24]. Five distinct functional domains can be identified in the ER-α protein moving from the N-terminus to the C-terminus, namely, the N-terminal domain (NTD), the DNA-binding domain (DBD), the D domain, the ligand-binding domain (LBD), and the F domain. The crystal structure of DBD and LBD have been determined by crystallographic studies [25,26,27]. Besides the natural ligand E2, several molecules have been identified that can interact with ER-α, providing either agonist or antagonist effect, with tamoxifen and fulvestrant representing the prototype for selective estrogen receptor modulators (SERM) and selective estrogen receptor degraders (SERD), respectively [28]. Several of these receptor–ligand complexes have been also studied at the structural level, leading to the identification of the main conformational changes involved in the activation and inactivation of the receptor [24] and providing a strong background to predict the effect of new ligands based on molecular docking and dynamic simulations.

In this study, integrating bioinformatics approach with cell biology and pharmacological methods, we demonstrated that mitotane directly bounds ER-α as a direct agonist, inducing cell proliferation of the ER-α positive breast cancer cell line MCF-7 cells. These results provide the biological and pharmacological basis of the clinically observed estrogen-like effect of mitotane.

## 2. Material and Methods

### 2.1. Ligand Docking Experiments

To investigate the interaction between mitotane and ER-α and whether this ligand would act as an agonist or antagonist, we compared the behavior of this simulated ligand-protein complex with those containing bisphenol A (BPA), bisphenol C (BPC), and E2. Compared to E2, the natural receptor ligand, or tamoxifen, a popular inhibitor, BPA and BPC are two small chemical compounds structurally similar to mitotane (Supplemental Figure S1) and known to interact with ER-α. Moreover, the mechanism of interaction between these two molecules and ER-α has been described in detail previously, and the analysis of crystal structures demonstrated how BPA acts as a weak agonist, while BPC acts as an antagonist [29]. Thus, these ligands were used as reference models for agonist and antagonist behavior in molecular dynamic simulations.

Starting from the crystal structure of the ER-α-E2 complex (1qku [25]), we first removed the natural ligand from the structure to generate a structure representing the apo form of the receptor and used this as the target structure in ligands docking. We simulated protein-ligand complexes between this target and four possible ligands, namely, E2, BPA, BPC, and mitotane. The structures of the ligands were obtained from DrugBank (https://go.drugbank.com/ accssed on 20 March 2018). Molecular docking was performed using the SwissDock platform (http://www.swissdock.ch/ accssed on 20 March 2018), which uses calculations performed in the CHARMM force field with EADock DSS [30]. Docking was performed allowing 3 Å side-chain flexibility and with a fixed target region of 20 Å with center at (X = 104, Y = 14, Z = 23). For each ligand, the predicted complexes were ranked according to their FullFitness value [31], and the top-ranking model was selected for molecular dynamics simulations.

### 2.2. Molecular Dynamics

Molecular dynamics (MD) simulations of the protein were performed by means of package GROMACS 2016 [32]. The initial structures of all the ER-α ligand complexes were taken from the output of the docking procedures. The force field amber99-sb was used, with the PME method for the Coulomb interactions and a Lennard–Jones potential with a cutoff of 10 Å for the short-range interactions. The initial structure was completed by the addition of hydrogens and solvated with TIP3P water in a simulation box with a minimum distance of 10 Å between solute and box boundaries. Na^+^ and Cl^−^ ions were added to reproduce a salt concentration of 150 mM and to neutralize the system. The system was first subjected to energy minimization, and then random velocities were assigned from a Maxwell distribution at the chosen temperature, and equilibration at constant volume for 100 ps, and at constant pressure for 100 ps was performed before the production run in the NPT ensemble. The temperature was kept constant by velocity rescaling with a characteristic time of 0.1 ps. The pressure was controlled using the Parrinello–Rahman method with a time constant of 1 ps and compressibility of 4.5 × 10^−5^ bar^−1^.

Force-field parameters for the ligand molecules BPA, BPC, tamoxifen, E2, and mitotane were obtained from the AMBER GAFF set of parameters [33] for covalent and nonpolar interactions, and by means of the RESP procedure for the partial charges. Quantum mechanical computations were executed by means of GAMESS [34].

Free energy perturbation calculations were executed by varying first the atomic partial charges of the ligand from their original values to zero in 5 steps and then varying the Van der Waals interactions parameters of the ligand from their original values to zero in 12 steps. The final value of free energy difference was obtained by numerical integration, through the trapezoidal rule, of the derivative of the Hamiltonian with respect to the perturbation parameter. For each case, the final value of free-energy variation was determined as the average of four independent calculations.

Free energy calculations using the MMGB/SA method were performed by means of a program developed by us for the molecular mechanics and generalized Born contributions, and by means of the Gromacs routines for the solvent accessible surface area.

### 2.3. Cell Treatment

MCF-7 cell line was purchased by American Type Culture Collection (LGC Standards, Sesto San Giovanni, Milan, Italy) and cultured as described in Fragni et al. [35]. The ER-, PgR-, Her2- MDA-MB-231 cell line, used as the internal negative control, was purchased by American Type Culture Collection (LGC Standards, Sesto San Giovanni, Milan, Italy) and cultured as suggested by the manufacturer. Media and supplements were supplied by ThermoFisher Scientific (Waltham, Massachusetts, USA). Cells were tested for mycoplasma and authenticated by BMR Genomics (Padova, Italy). Cells were plated in 24-well plates (1 × 10^4^ cells/well) for viability assay and in 6-well plates (3.2 x 10^4^ cells/well) for cell proliferation assay in the complete medium. Then, 24 h after being seeded, the medium was switched to a medium containing dextran-treated charcoal-stripped serum (CSS medium), and cells were treated with increasing concentrations of mitotane (3–24 µM) for 3 days, according to the calculated doubling time. For experiments with tamoxifen, cells were pretreated for 1 h with increasing concentrations (1–10 µM) of the SERM, according to the experimental protocol, and then 6 µM mitotane was added and treated for 3 days. Mitotane and tamoxifen were purchased from Selleckchem Chemicals (DBA Italia, Segrate (MI), Italy) and were both dissolved in DMSO. Cell exposure to DMSO alone did not modify the cell viability.

### 2.4. Cell Viability and Cell Proliferation Experiments

Cell viability was evaluated by 3-(4.5-dimethyl-2-thiazol)2.5-diphenyl-2H-tetrazolium bromide (MTT) dye reduction assay as previously described [36]. Absorbance was measured by an EnSight Multimode Plate Reader (PerkinElmer Italia, Milan, Italy) at 570 nm.

The cell proliferation rate was evaluated with a TC20 automated cell counter (Bio-Rad, Segrate, Milan, Italy). Briefly, cells were grown in 6-well plates, dislodged by trypsinization, and suspended in a culture medium, followed by trypan blue dilution (1:2). The parameter settings were established according to the manufacturer’s instructions. Ten microliters of the mixture were loaded into the opening of the TC20 counting slide. The cell counter automatically detects the presence of the counting slide and initiates the count. The gating 4–16 µm was selected as optimal for MCF-7 cells, based on preliminary validation studies.

### 2.5. Transient Silencing of ER-α

MCF-7 cells (4 × 10^4^ cells/well) were seeded in a 24-well plate in the complete medium. Briefly, 24 h after seeding, cells were transfected with two different ER-α siRNA sequences (50 nM) and scrambled siRNA sequences (40 nM) using lipofectamine RNAiMax (Invitrogen, Carlsbad, CA, USA) following the supplier’s instructions. Human ER-α siRNA (s4823 and s4824) and scrambled siRNA were purchased from Ambion (Life Technologies, Carlsbad, CA, USA). The siRNA sequences were, respectively, siRNA ER-α s4823 S: ACAUCAUCGGUUCCGCATT AS: UGCGGAACCGAGAUGAUGUAG; siRNA ER-α s4824 S: CAGGCACAUGAGUAACAAATT; AS: UUUGUUACUCAUGUGCCUGAT. Cells were then incubated at 37 °C for 6 h. After removal of the transfection medium, cells were maintained in CSS medium for 18 h. Then, 6 µM mitotane was added, and cells were treated for 3 days.

### 2.6. Western Blot Analysis for ER-α Expression

The expression level of ER-α protein was determined by the immunoblotting analysis to ensure the knockdown of the target genes at 24–48–72 h after silencing. Cells (3 × 10^6^ cells) were lysed in ice-cold RIPA buffer with a complete set of protease and phosphatase inhibitors (Roche, Milan, Italy). Protein concentration was measured using the Bradford Protein Assay and 40 µg of the whole lysate was separated by electrophoresis on a 7.5% Bis–Tris gel and electroblotted to a nitrocellulose membrane, following the manufacturer’s instructions. Membranes were reacted using a primary antibody against human-ER-α raised in mouse (final concentration: 0.8 µg/mL; Invitrogen, Carlsbad, CA, USA). A rabbit polyclonal antibody directed against the N-terminal region of human β-Tubulin III (final concentration: 0.3 µg/mL; Merck, Darmstadt, Germany) was then applied to membranes to normalize the band intensity. Secondary HRP-labelled anti-rabbit and anti-mouse antibodies (final concentration: 0.08 µg/mL; Santa Cruz Biotechnologies, Heidelberg, Germany) were applied for 1 h at room temperature. The specific signal was visualized by the Westar ηc ultra 2.0 ECL (Cyanagen, Bologna, Italy). Densitometric analysis of the immunoblots was performed using the NIH ImageJ Software.

### 2.7. Statistical Analysis

The analysis of the data was carried out by the GraphPad Prism version 5.0 software (GraphPad Software, La Jolla, CA, USA), using the one-way ANOVA, with a post hoc test (Bonferroni’s test) for multiple comparisons, considering *p* < 0.05 as the threshold for a significant difference. Data are expressed as mean ± SEM of three independent experiments unless otherwise specified. Cytotoxicity and proliferation experiments were carried out at least three times, each point running in triplicate.

## 3. Results

### 3.1. Mitotane Docking on ER-α

**Figure 1:**
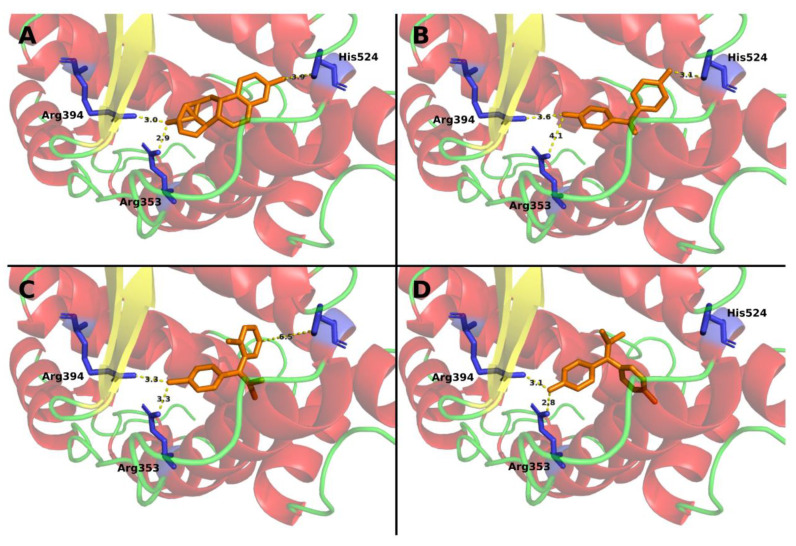
Docking of the different compounds in the binding pocket of ER-α. The predicted placement of ligand within the binding pocket of ER-α was predicted using SwissDock for E2 (**A**), BPA (**B**), mitotane (**C**), and BPC (**D**). Ligand is represented in orange and the three main residues involved in ligand interactions are marked in the structure in blue. When arranged in the binding pocket, the 2 agonist compounds, namely, E2 and BPA are placed within reasonable distance from the interacting residues, while the antagonist BPC assumes a different conformation, losing interaction with His524. Interestingly, mitotane seems to behave similarly to agonist compounds.

Ligand docking prediction using SwissProt resulted in four complexes including ER-α and E2, BPA, BPC, or mitotane with FullFitness of −646.3, −1079.8, −1070.9, −1057.5, respectively. In all complexes, the ligand was placed in the active site with a similar orientation, compared to the native ligand E2, showing that they can fit in the binding pocket in a correct configuration. Mitotane showed a similar configuration, compared to E2 (natural ligand) and BPA (weak agonist), and all three were placed at interaction distance from the key active site amino acids Glu353, Arg394, and His524 [24,37], while BPC (antagonist) bound in a different configuration, unable to interact with His524 (Figure 1). These four complexes were used as starting points for molecular dynamics simulations to compare the behavior of the mitotane-ER-α complex with those containing BPA (weak agonist), BPC (antagonist), and E2 (natural ligand).

### 3.2. Molecular Dynamics

The computational study had two aims, i.e., first to determine whether mitotane acts as an agonist and second to evaluate the binding affinities of mitotane and other ligands with ER-α comparatively.

#### 3.2.1. Mitotane in Molecular Dynamics Simulations Acts as an Agonist

Molecular dynamics simulations of the ER-α dimer, in complex with BPA, with BPC, and with mitotane were performed at physiological temperature, 310 K. In all cases, the initial structure, taken from the docking procedures, was in the agonist form, and no significant motion of helix H12 was observed during the simulations. However, the time scale for such motions may be much longer than the accessible simulation times. Therefore, in order to accelerate the dynamics, the temperature was raised by 100 K, up to 410 K. For each complex, eight independent runs of 50 ns were executed, giving an aggregated simulation time of 400 ns. Moreover, the trajectories of the two subunits of each dimer were concatenated, and thus, a final amount of 800 ns was obtained for each monomeric complex. A complete displacement of helix H12 from the closed to the open position was observed in one case in the MD simulations of BPC (see Supplemental Figure S2). A partial displacement of helix H12 was also observed for a short time in the simulations of BPA, but it was never observed in the simulations of mitotane.

**Figure 2:**
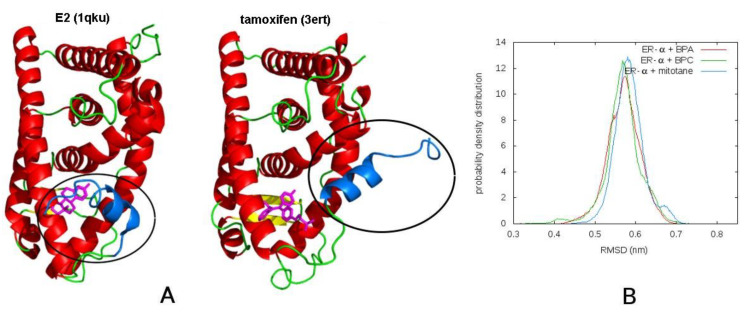
Conformation of active and inactive ER-α and comparison with MD results. (**A**) the active conformation of the ER-α receptor is represented on the left based on the 1qku structure, containing the receptor in complex with the natural ligand E2. The inactive form is represented on the right based on the 3ert structure, in complex with the tamoxifen inhibitor. The mobile H12 helix, crucial in the activation of the receptor, is represented in blue. The inhibitor compound displaces the H12 helix from the binding pocket, disrupting the cofactors binding surface; (**B**) probability density distribution of the RMSD of the backbone atoms with respect to the 3ert structure, obtained from the molecular dynamics simulations of the complexes of ER-α with BPA (red curve), with BPC (green curve) and with mitotane (blue curve).

A quantitative comparison of the tendency to adopt the antagonist conformation is given by the root mean square deviation (RMSD) of backbone atoms of the monomer with respect to the crystal structure with PDB code 3ERT. The distributions of RMSD values resulting from MD simulations at 410 K (Figure 2) clearly indicate that the curve obtained for the complex with BPC extends to lower values with respect to those with BPA and mitotane, showing a slightly higher similarity to the reference antagonist structure 3ERT. The curve for mitotane, on the contrary, is shifted toward higher values with respect to both BPA and BPC, and this suggests classifying mitotane as an agonist ligand.

#### 3.2.2. Binding Free Energies from MD Calculations

The binding free energies of ligands BPA, BPC, tamoxifen, E2, and mitotane to ER-α were estimated by two independent computational methods based on MD simulations: free energy perturbation (FEP, see Supplemental Scheme S1 for details), and MMGB/SA [34]. Both methods take as a starting point the structures generated by the docking procedure, except for the complex with tamoxifen for which a crystal structure is available. Therefore, the initial phase of the interaction, involving the access of the ligand to the binding site, is not taken into account. This point will be further discussed later.

Since the aim of the study is a comparative analysis of the five ligands, the difference of the binding free energy of each ligand with respect to that of E2 is reported in Table 1, where a positive difference corresponds to a weaker binding.

The results show that BPA, BPC, and mitotane have weaker binding with respect to E2, while for tamoxifen, one method gives a negative value, and the other method gives a positive value, suggesting that its binding energy could be comparable to that of E2.

As pointed out before, this analysis does not include the phase of access of the ligand to the binding site and the possible associated conformational changes. Indeed, the results obtained here are in qualitative agreement with experimental data concerning the kinetic dissociation constants, kd, reported in Table 1 of ref. [38] for some of the ligands under study, namely, BPA, E2, and tamoxifen. However, in ref. [38], the equilibrium dissociation constant, KD, of tamoxifen is much larger than that of E2 as a consequence of a much lower kinetic association constant, ka, due to the large size of tamoxifen, which requires a conformational change of helix H12 to enable its access to the binding site. In this respect, BPA and E2 have similar behavior, as shown by the equal value of their kinetic association constants reported in ref. [38]. The case of mitotane can be compared to that of BPA by observing that its relative binding energy, as shown in Table 1, is estimated to be lower (stronger binding, lower kd), while, as shown above, mitotane affects the dynamics of helix H12 less than BPA. This suggests that the equilibrium dissociation constant KD of mitotane should be lower than that of BPA and thus lower than that of tamoxifen.

### 3.3. Mitotane Induced an Increase of MCF-7 Cell Viability and Proliferation via ER-α Stimulation

**Figure 3:**
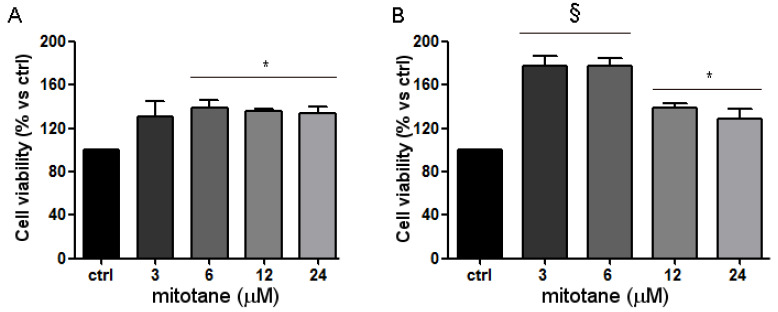
Effect of mitotane on MCF-7 cell viability and proliferation. ER-α-positive MCF-7 cell line was treated with increasing concentrations of mitotane as described in Methods: (**A**) cell viability was measured by MTT assay. Data are expressed as percentage vs. untreated cells ± SEM. * *p* < 0.05; (**B**) cell proliferation rate was estimated by direct count, with trypan blue discrimination. ^§^ *p* < 0.0001 vs. untreated cells; * *p* < 0.01 vs. untreated cells. Data are expressed as percentage vs. untreated cells ± SEM. Results are the mean of three independent experiments run in triplicate.

Exposure of breast cancer (BC) MCF-7 cell line to increasing concentrations of mitotane (3–24µM) for three days, led to a concentration-dependent increase of cell viability and cell proliferation rate (Figure 3). In particular, the increase of viability reaches its maximum at 6 µM mitotane, with stimulation of 50% ± 11.36% over untreated cells (Figure 3A) that was maintained up to 24 µM. With the same trend, mitotane also induced a concentration-dependent increase of the cell proliferation rate, as reported in Figure 3B.

**Figure 4:**
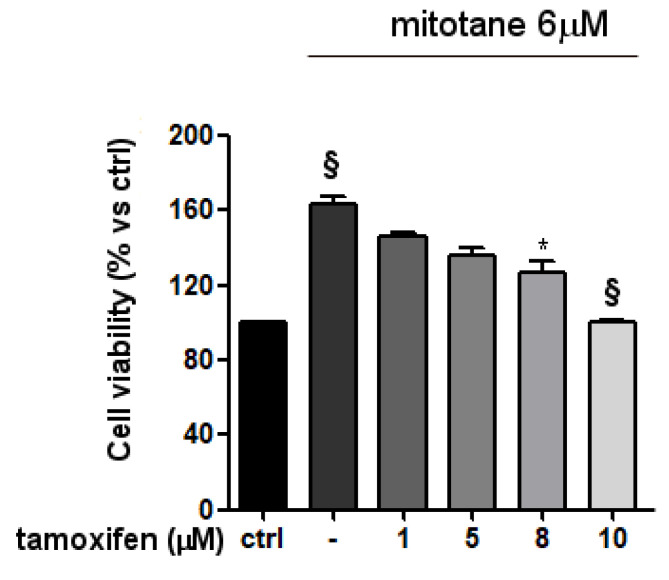
Tamoxifen antagonizes mitotane effect on MCF-7 cell viability. ER-α-positive MCF-7 cell line was pretreated for 1 h with increasing concentrations of tamoxifen as above described, then mitotane 6 µM was added to the medium. Cell viability was measured by MTT assay after three days of treatment. Results are expressed as percentage of viable cells ± SEM vs. untreated cells (ctrl). Data are the mean of three different experiments, each point run in triplicate. ^§^ *p* < 0.0001 vs. ctrl; * *p* < 0.001 vs. mitotane 6 µM treated cells without tamoxifen.

To evaluate whether the mitotane effect on the MCF-7 cell line was due to the binding with ER-α, cells were treated with 6 µM mitotane in the presence of increasing concentrations of the SERM tamoxifen and analyzed for cell viability. As shown in Figure 4, tamoxifen reduced in a concentration-dependent manner the increased cell viability induced by mitotane in the MCF-7 cell line. The triple-negative BC MDA-MB-231 cell line, lacking ER-α, did not modify its cell viability when exposed to increasing concentrations of mitotane (Supplemental Figure S4).

**Figure 5:**
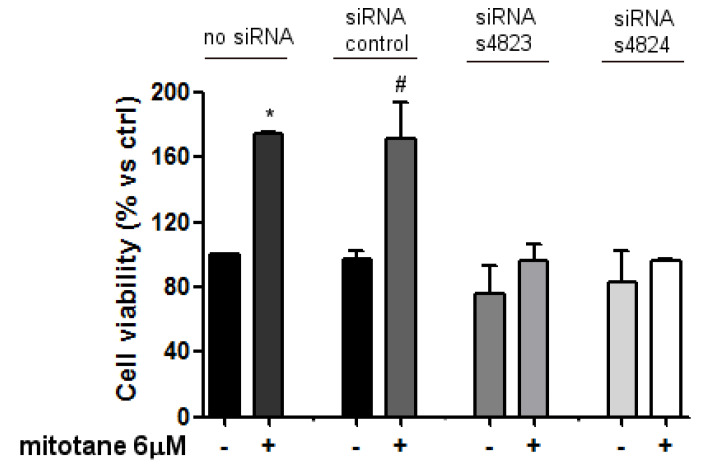
Effect of ER-α silencing on mitotane activity in MCF-7 cell line. Untreated cells, siRNA scramble, and siRNA s4823/s4824-treated MCF-7 cells were exposed to mitotane 6 µM for three days. Cell viability was measured by MTT assay. Results are expressed as percentage of viable cells ± SEM vs. ctrl. Data are the mean of three different experiments, with each point running in triplicate. * *p* < 0.01 vs. untreated cells (ctrl); # *p* < 0.01 vs. siRNA scramble-treated cells.

The capability of mitotane to bind ER-α was confirmed using an RNA interference approach, silencing the ER-α gene. Following 24 h of transfection, both siRNA ER-α efficiently downregulated ER-α protein level (−63.52% for siRNA ER-α s4823 and −58.64% for siRNA ER-α s4824), and this effect was maintained up to 3 days (Supplemental Figure S3). The effect of ER-α knockdown on mitotane-induced MCF-7 cell viability was then investigated in cells exposed to both siRNA ER-α or si-control for 24 h and then treated with 6 µM mitotane. Silencing MCF-7 cells by both siRNAs significantly prevented the stimulatory effect of mitotane (Figure 5), strongly suggesting that mitotane was able to bind ER-α, inducing a stimulatory growth effect.

## 4. Discussion

Sixty years after the introduction of mitotane in the medical treatment of ACC, the whole spectrum of its pharmacological activities remains elusive. The main effect of mitotane in ACC is the induction of tumor cell death by apoptosis and/or necroptosis [39] and the on-target result of toxic disruption of adrenocortical steroidogenesis induced by mitotane remains the reduction of glucocorticoid excess, and other endocrine effects are considered off-target results. Among these, endocrine effects attributable to enhanced estrogen activity have been only partially studied, and the main conclusion of previous studies is that mitotane has an estrogenic effect that is ER-α dependent and mediated by mitotane-dependent increased hepatic SHBG synthesis [15]. However, the nature of the interaction between mitotane and ER-α and its downstream biological and pharmacological consequences are not known.

In this work, we provided robust molecular, mechanistic, and biological evidence for a direct interaction of mitotane and ER-α.

The study of crystal structures of complexes with agonist and antagonist molecules identified the helix 12 (H12, residues 532–552) as the most important structural element of each LBD monomer, acting as a molecular switch between the active and inactive conformation of the receptor with “flip-flop” mechanism [24,25,26,40]. After ligand interaction, the H12 helix undergoes a conformational change, assuming either an active conformation, where it is packed against helixes H3, H5/6, and H11 to close the binding site and create a surface essential for coactivators binding, or an inactive conformation, where it is displaced from the binding site and located in a groove formed by H3 and H5 [24,37,39]. As a consequence, the conformation of helix H12 can be used to assess the ability of a ligand to act as an agonist or antagonist in molecular dynamics simulations [40].

This structural analysis was the basis for the design and implementation of the computational part of our study aimed at comparing mitotane with other known ligands. The structures of the complexes produced by the docking calculations provided the first hint on the behavior of mitotane because of the similarity in the predicted receptor–ligand configuration for E2, the natural ligand, and mitotane. Using molecular dynamics simulations, we compared the possible conformational changes induced by mitotane and two small molecules with similar structures acting as a receptor agonist (BPA) or antagonist (BPC) [29]. These simulations showed, through the analysis of key structural indicators, that the effects of mitotane on ER-α are comparable to those of agonist ligand BPA. The significance of the result is stressed by the fact that an antagonist behavior was observed for BPC under the same conditions. Finally, binding free energy calculations, combined with previous experimental data, suggested that mitotane has a higher affinity to ER-α, as compared to tamoxifen, but lower with respect to E2.

We then verified in a biological system the results of our computational model, which indicated the capability of mitotane to bind ER-α as an agonist, by testing the hypothesis that mitotane could exert a direct estrogenic biological activity in the ER-α-positive MCF-7 cell line. Results confirmed the bioinformatics finding: as expected from a drug stimulating ER-α in cells derived from breast, mitotane was able to induce the cell proliferation rate and to increase cell viability at experimental concentrations that were even lower than of those reported in the clinic. The involvement of ER-α in eliciting this effect was demonstrated by both pharmacological and molecular approaches. According to the initial experimental hypothesis, mitotane induced an increase in cell viability, which was reduced when cells were exposed to mitotane in the presence of tamoxifen. In addition, the effect induced by mitotane was completely reverted by silencing ER-α, while and triple-negative breast cancer (BC) cell line MDA-MB-231. These results represent the first demonstration of direct agonistic interaction between mitotane and ER-α at the molecular and cellular levels.

Thus, besides the already known increased synthesis and binding capacity of SHBG in mitotane-exposed ACC patients, here we provided robust evidence demonstrating that the estrogen-like effects observed in the clinic are due to the direct capability of mitotane to bind and activate ER-α. These results supported the rationale to antagonize these side effects with drugs such as the SERM tamoxifen or the SERD fulvestrant, added to the mitotane therapy. This point, however, needs to be carefully addressed since, although fulvestrant did not report significant drug–drug interaction [41], the chronic administration of this combination may result in a drug–drug metabolic interaction between tamoxifen and mitotane, due to the CYP3A4-mediated tamoxifen metabolism [42] and the well-known CYP3A4 inducer effect of mitotane [39]. From a pharmacological point of view, however, the interaction may be more complex than described above. Indeed, hepatic metabolism of tamoxifen generates about 20 different metabolites, among which Z-endoxifen (through CYP2D6) and 4-hydroxytamoxifen (mainly through CYP2D6, but also CYP2C9, CYP2C19, and CYP3A4) present 30–100-fold higher activity than the parental compound [43]. Thus, genetic polymorphisms and drug–drug interactions affecting CYP isoenzymes activity may impact plasma concentrations of active metabolites with possible consequences for the expected therapeutic outcome.

In conclusion, we here demonstrated that mitotane directly binds ER-α as an agonist and suggested that SERM or SERD could be clinically useful to antagonize the estrogen-like mitotane-induced effects. This latter point, however, needs to be demonstrated in a dedicated clinical study, taking into account the complex pharmacokinetics of mitotane.

## Figures and Tables

**Table 1:** Binding free energies in kcal/mol of different ligands with ER-α expressed as differences with respect to the value of E2.

Method	BPA	BPC	Mitotane	Tamoxifen
MMGBSA	12.8	10.1	9.9	−4.6
FEP	11.0	10.4	8.4	2.9

## Data Availability

Not applicable.

## Supplementary Materials

The following are available online at https://www.mdpi.com/article/10.3390/biomedicines9060681/s1, Scheme S1: Thermodynamic cycle used to compute the binding free energy of ligand L to protein P, Figure S1: Chemical structure of the four analyzed compounds, Figure S2: Structure of the complex of ER-α with BPC taken from molecular dynamics simulations at 410 K, where helix H12 (highlighted in green) has an antagonist conformation, Figure S3: Estrogen receptor α silencing was detected by Western blot assay, Figure S4: Effect of mitotane on MDA-MB-231 cell viability.

## Notes

The views expressed are those of the authors and not necessarily those of the NIHR or the Department of Health and Social Care. M.T. and A.A. are enrolled in the PhD Program on Precision Medicine, University of Brescia.
