# Supplementary material for: Estrogen-Like Effect of Mitotane Explained by Its Agonist Activity on Estrogen Receptor-α
Source: Biomedicines. 2021 Jun 16;9(6):681. doi: 10.3390/biomedicines9060681 (PMC8235434; doi:10.3390/biomedicines9060681)
Supplement: Supplementary file 1 [file biomedicines-09-00681-s001.zip › biomedicines-1259771-supplementary.pdf]

biomedicines

Article

Estrogen-Like Effect of Mitotane Explained by Its Agonist Activity on Estrogen Receptor-α

Elisa Rossini [1,†], Edoardo Giacopuzzi [2,3,†] Fabrizio Gangemi [4,†], Mariangela Tamburello [1], Deborah Cosentini [5], Andrea Abate [1], Marta Laganà [5], Alfredo Berruti [5], Salvatore Grisanti [5,‡], and Sandra Sigala [1,*,‡]

Supplementary materials

Supplementary Data, Supplemental Materials, and Methods Free energy perturbation: Binding free energies between ER-α and a ligand were calculated by means of the thermodynamic cycle shown in Supplemental Scheme 1, where P indicates the protein, L the ligand, and $L_0$is the artificial state of the ligand in which its interactions with the rest of the system are set to zero. The free energy difference DG between the bound state and the unbound state is given by $DG = DG_1 + DG_2 - DG_3$, where DG_1 and DG_3 were calculated by the free-energy perturbation technique, as described below, while DG_2 vanishes, due to the absence of interactions of $L_0$with all the other molecules in the system. In order to determine DG_1 and DG_3, a series of simulations were performed, both for the complex and for the ligand alone, in which the interactions of the ligand with the rest of the system are gradually taken to zero by variation of a parameter in the Hamiltonian.

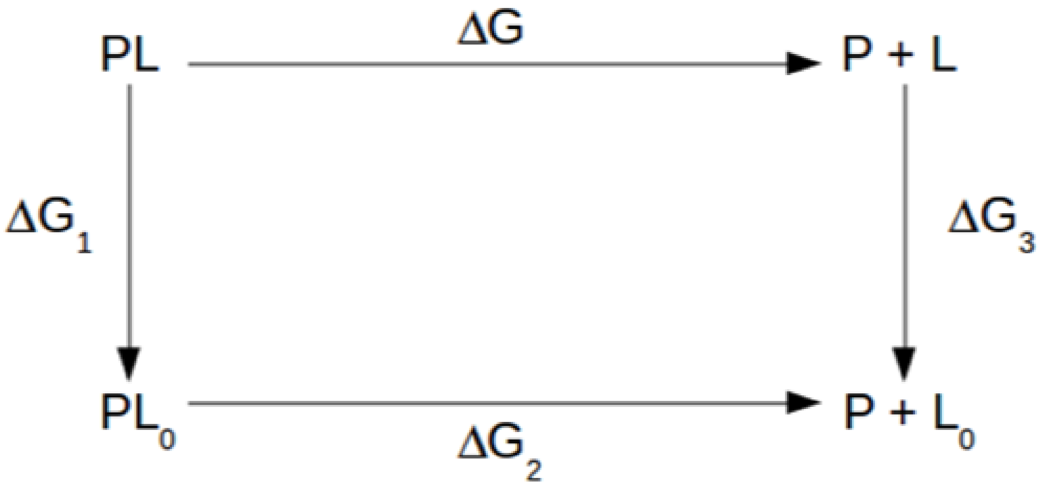

Scheme 1. Thermodynamic cycle used to compute the binding free energy of ligand L to protein P. In this scheme, PL denotes the bound state while P + L denotes the unbound state, and L_0denotes the ligand with all noncovalent interactions removed.

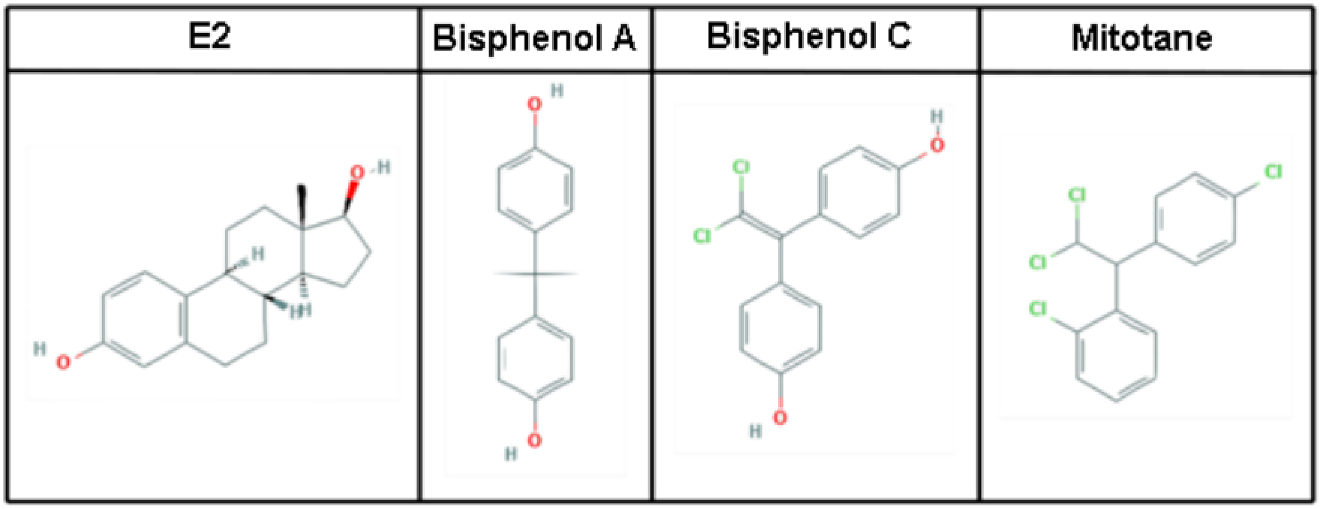

Figure S1. Chemical structure of the four analyzed compounds. The plain chemical structure of E2, BPA, BPC, and mitotane is reported. The mitotane molecule has a similar size and structure, compared to the BPA agonist and BPC antagonists.

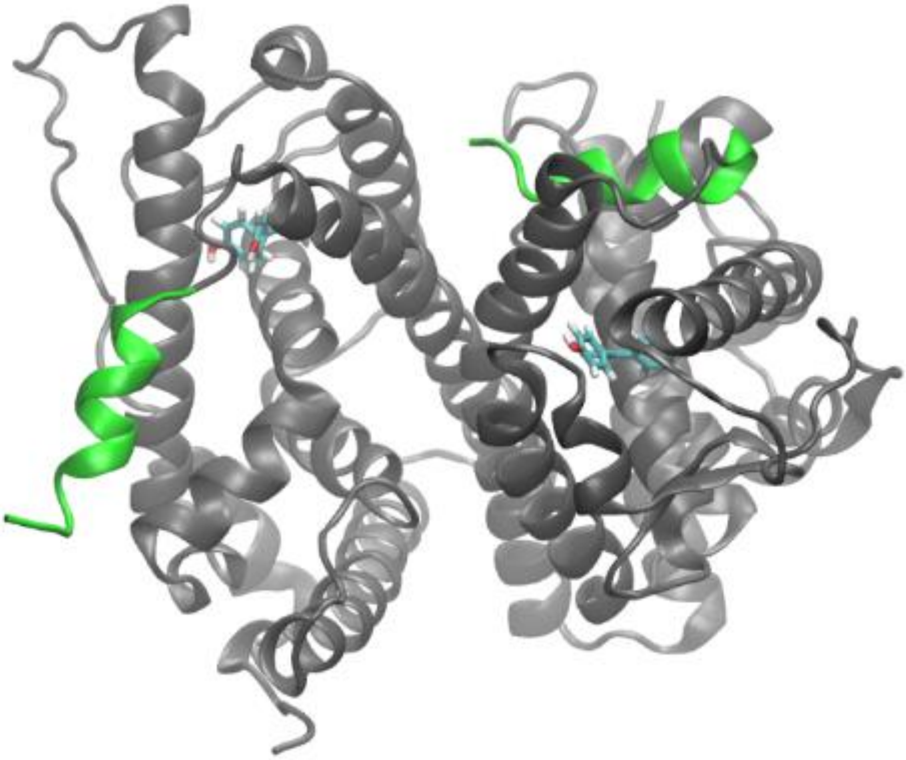

Figure S2. Structure of the complex of ER-$\alpha$with BPC taken from molecular dynamics simulations at 410 K, where helix H12 (highlighted in green) has an antagonist conformation.

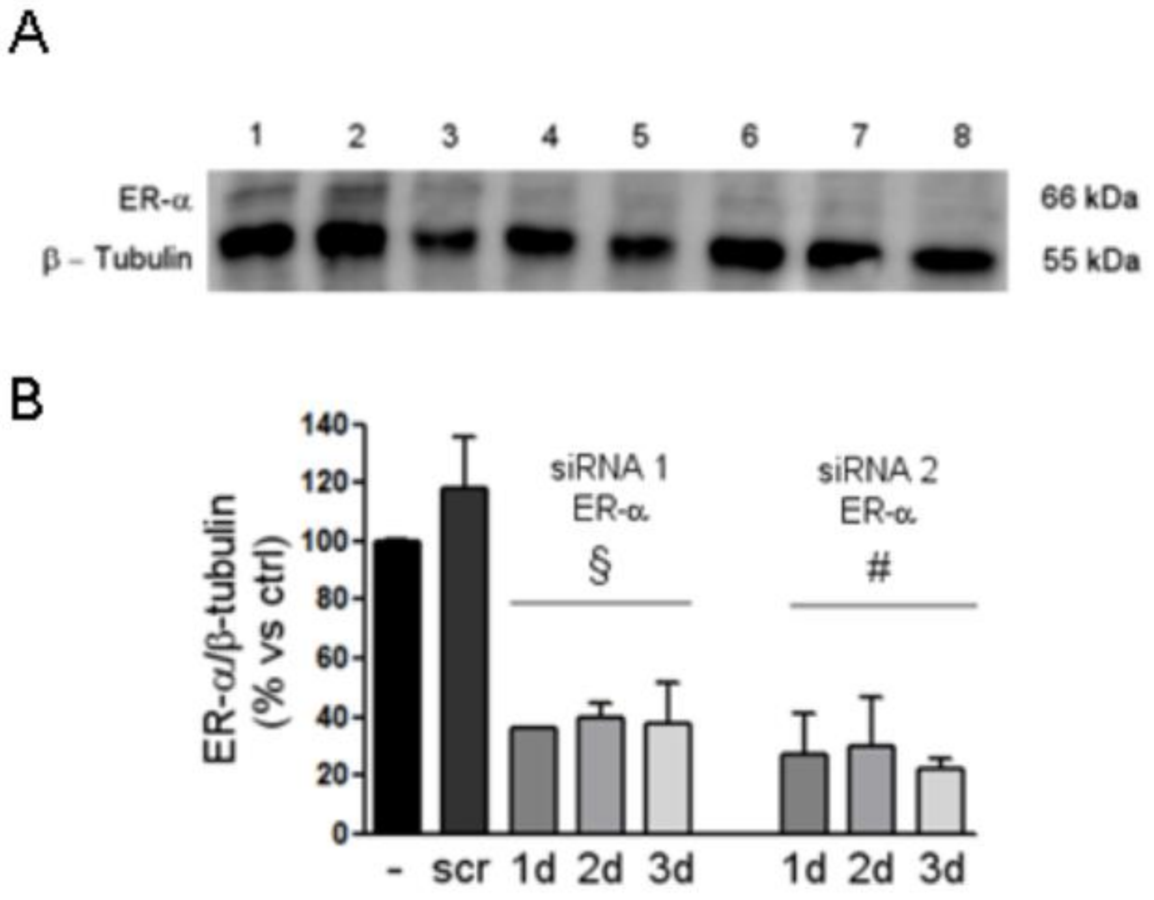

Figure S3. Estrogen receptor *α*silencing was detected by western blot assay: (**A**) Western blot was conducted, as described in Methods; human β-tubulin was used as internal control. A representative blot is shown: untreated (1); scramble (2); siRNA 1: s4823 24h (3); s4823 48h (4); s4823 72h (5); siRNA 2: s4824 24h (6); siRNA s4824 48h (7); s4824 72h (8); (**B**) densitometric analysis was conducted using NIH ImageJ Software. Results are expressed as percent vs ctrl ± SEM. §P< 0.05 vs. untreated cells (ctrl); #P< 0.001 vs. untreated cells.

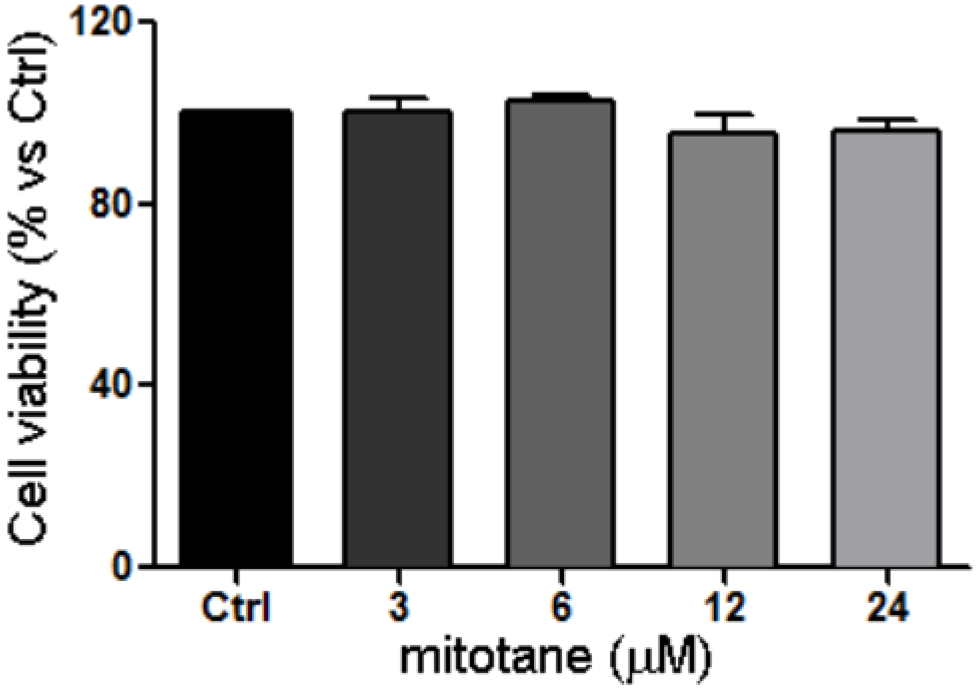

Figure S4. Effect of mitotane on MDA-MB-231 cell viability. Triple-negative breast cancer cell line MDA-MB-231was treated with increasing concentrations of mitotane as described in Methods. Cell viability was measured by MTT assay. Data are expressed as percentage vs. untreated cells ± SEM.
